# A novel high-content screening approach for the elucidation of *C. jejuni* biofilm composition and integrity

**DOI:** 10.1186/s12866-020-02062-5

**Published:** 2021-01-04

**Authors:** Matthew V. X. Whelan, Jeremy C. Simpson, Tadhg Ó Cróinín

**Affiliations:** 1 School of Biomolecular and Biomedical Science, University College Dublin, Belfield, Dublin 4, Ireland; 2 School of Biology and Environmental Science, University College Dublin, Belfield, Dublin 4, Ireland

## Abstract

**Background:**

*Campylobacter jejuni* is the leading cause of bacterial gastroenteritis worldwide and the main source of infection is contaminated chicken meat. Although this important human pathogen is an obligate microaerophile, it must survive atmospheric oxygen conditions to allow transmission from contaminated chicken meat to humans. It is becoming increasingly evident that formation of biofilm plays a key role in the survival of this organism for extended periods on poultry products. We have recently demonstrated a novel inducible model for the study of adherent *C. jejuni* biofilm formation under aerobic conditions. By taking advantage of supercoiling mediated gene regulation, incubation of *C. jejuni* with subinhibitory concentrations of the Gyrase B inhibitor novobiocin was shown to promote the consistent formation of metabolically active adherent biofilm.

**Results:**

In this study, we implement this model in conjunction with the fluorescent markers: TAMRA (live cells) and SytoX (dead cells, eDNA) to develop a novel systematic high-content imaging approach and describe how it can be implemented to gain quantifiable information about the integrity and extracellular polymeric substance (EPS) composition of adherent *C. jejuni* biofilm in aerobic conditions. We show that this produces a model with a consistent, homogenous biofilm that can be induced and used to screen a range of inhibitors of biofilm adherence and matrix formation.

**Conclusions:**

This model allows for the first time a high throughput analysis of *C. jejuni* biofilms which will be invaluable in enabling researchers to develop mechanisms to disrupt these biofilms and reduce the viability of these bacteria under aerobic conditions.

**Supplementary Information:**

The online version contains supplementary material available at 10.1186/s12866-020-02062-5.

## Introduction

*Campylobacter jejuni* is the leading cause of bacterial gastroenteritis and a significant health burden across the world, with over 550 million cases reported each year. It is becoming increasingly evident that formation of biofilm is a key aspect of *C. jejuni* virulence, in particular as a stress response mechanism to explain the conundrum of the survival of this highly fastidious organism within the food chain, from farm to fork [1]. Despite this, there remains much to be discovered about the regulation and composition of biofilm formation in *C. jejuni*. Biofilm formation is influenced by a variety of environmental factors including oxygen availability, temperature, osmolarity and availability of nutrients [2, 3]. *C. jejuni* is capable of forming three types of biofilm, (i) a structure adherent to an abiotic surface, (ii) self/mixed-aggregates floating within a liquid culture or (iii) a pellicle layer at the liquid air interface [4]. *C. jejuni* benefits from enhanced fitness by formation of adherent biofilms in the presence of stressful environmental conditions.

*Campylobacter* biofilm formation has been shown to be influenced by a broad range of factors including the stringent stress response (spoT, Ppk1*,* Ppk2*)* [5–7], molecular chaperones (EF-G, ClpP/A/X) [8], two-component regulatory systems (CprRS) [9], LOS structure and modification (waaf, lgtF, EptC) [10, 11], OMP profile (Peb4) [12], quorum sensing (*luxS*) [2], chemotaxis (FucP) and the oxidative stress response (PerR, CosR, CsrA) [8, 13]. *Campylobacter* adhesins (CadF) and moonlighting proteins (Dps) have been shown to be required for initiation of biofilm formation on an abiotic surface [14, 15]. Flagellar integrity and motility play key roles in adherence to abiotic surfaces, during chicken colonisation, biofilm formation on human ileal tissue and formation of bacterial cell-cell connections facilitating net-like structures during biofilm maturation [8, 16–18]. Biofilm formation can also be induced by host and environmental factors such as bile salt sodium deoxycholate (DOC), which has been well documented for its role in increasing virulence factor expression, secretion and infection kinetics in *C. jejuni* [9, 19]. There is evidence suggesting that induction of *C. jejuni* biofilm on chicken meat may facilitate survival [20]. *C. jejuni* strain NCTC11168 develops adherent biofilms more rapidly when incubated aerobically rather than under ideal microaerobic gas conditions [3]. Recent work has continued to implicate the role of the oxidative stress response regulon in modulation of biofilm formation when *C. jejuni* is exposed to a high oxygen environment [21, 22].

The extracellular polymeric substance (EPS) is a vital component of bacterial biofilms, accounting for as much as 90% of the biofilm mass [23]. The self-produced extracellular matrix assists the bacteria by providing structural support, antimicrobial resistance, sequestering of nutrients and preventing dehydration [24]. Initially an extracellular fiber-like material in the biofilm produced by NCTC11168 was reported, structurally resembling a net like matrix [8]. Sensitivity of *C. jejuni* biofilm to DNase I treatment revealed extracellular DNA (eDNA) as the likely major constituent in the extracellular matrix (ECM) of adherent *C. jejuni* biofilms [1, 24]. eDNA release is reported to be independent of functional flagella, insufficient alone to allow for biofilm formation (requiring flagellar mediated adherence) and likely released due to an as yet undefined autolytic mechanism, which has been described in other bacteria such as *P. aeruginosa* [1, 8, 9, 25]. A composition assay carried out on the biofilm of six strains found that 1 mg/mL proteinase K (protein degradation) promoted almost total degradation of established biofilm, while treatment with 10 mM sodium metaperiodate (carbohydrate oxidation) had little to no effect on biofilm structure [26]. Interestingly, in this same study, three antimicrobial agents used to treat chicken during production, namely paracetic acid (0.8%), sodium hyperchlorite (1%) and chlorohexidine (1%) were screened for their ability to eliminate adherent biofilm of 30 *C. jejuni* strains, and although they were effective in some cases, 30% of strains presented with resistance to at least one of the chemical agents while residing within adherent biofilm.

The overarching mechanisms of *C. jejuni* biofilm formation remain elusive. This has been confounded by the relatively low levels of biofilm formed, the lack of information on the viability of the biofilm and the relative low throughput of conventional confocal microscopy in combination with viability staining of biofilms. The lack of sensitivity of conventional assays such as crystal violet staining combined with the extreme heterogeneity of phenotypes observed amongst *C. jejuni* strains has also been a stumbling block. A significant issue in the field has been the lack of a rapid, highly sensitive, systematic approach to screen *Campylobacter* biofilm formation. Using a fluorescence microscopy approach, we recently demonstrated that relaxation of negative DNA supercoiling promoted an adherent viable biofilm forming phenotype under aeration [27]. Using a combination of microscopy and viability staining with TAMRA, a rhodamine-based dye which is cell permeant and upon entering the cytosol is enzymatically crosslinked by bacterial esterases, preventing the dye from escaping the intact bacterial cell, providing a bright indicator of living bacteria, we were able to detect adherent biofilm phenotypes which were not distinguishable using the crystal violet assay [28, 29]. The observed induced biofilm forming phenotype correlated with increased invasion of intestinal epithelial cells and killing efficiency of *Galleria mellonella* larvae for the strain NCTC11168 [27, 30]. This work revealed the potential of using novel microscopy approaches in conjunction with fluorescent viability stains to detect the presence and fluctuations of *C. jejuni* biofilms forming at levels which were not easily resolvable by conventional means.

The aim of this study was to adapt high-content screening approaches to quantitatively measure the effect of a variety of inhibitors of EPS integrity on the novobiocin-induced NCTC11168 biofilm under aeration. The challenge was to design a robust and quantitative approach to systematically analyse the structure and composition of the highly variable *Campylobacter* biofilm. Traditional microscopy approaches to study *Campylobacter* biofilm suffer from the limitations of low throughput and subjectivity of image acquisition. In this study, we used high-content screening microscopy in conjunction with TAMRA (live cell) and SytoX (dead cells, eDNA) biofilm labelling to systematically analyse the effect of a broad range of biofilm inhibitors on an induced aerobic biofilm phenotype of strain NCTC11168. Our experimental approach allows for the rapid quantification of the effect of a broad range of inhibitors on biofilm viability and structural integrity. This high-content screening approach to biofilm screening offers a broad range of applications, from rapid characterisation of isolates to screening potential antimicrobial and bactericidal chemical candidates to tackle the growing concern of medically relevant bacterial biofilms.

## Materials and methods

### Bacterial strains and growth conditions

*C. jejuni* NCTC11168 is the type strain of *C. jejuni* and a commonly used laboratory strain. *C. jejuni* were cultured on Mueller Hinton (MH) agar (Oxoid) at 37 °C under microaerobic conditions generated using Campygen gas packs (Oxoid). For liquid cultures, *C. jejuni* strains were equalised to specific optical densities in MH broth and incubated under microaerobic conditions at 37 °C, shaking at 200 rpm. *C. jejuni* stock cultures were maintained using MH broth (Oxoid) supplemented with 20% glycerol and stored at -80 °C. To relax DNA supercoiling levels and induce biofilm formation, strains were grown in the presence of 10 μg/ml novobiocin as previously described [27, 30].

### Optical biofilm inhibitor screening

Bacterial overnight cultures of NCTC11168 (Induced with 10 μg/mL novobiocin) were equalised to an OD600 of 0.1 in MH broth (supplemented with 10 μg/mL novobiocin) and 200 μL were seeded into all of the wells of a CellCarrier Ultra optical 96-well plate (PerkinElmer). The cultures were then incubated for 72 h at 37 °C aerobically (21% O_2_) to induce adherent biofilm formation. Each row of the 96-well plate was selected for addition of an inhibitor to be tested for its effect on biofilm integrity. After 72 h incubation, the adherent biofilms in all 96 wells were washed once with PBS in aseptic conditions followed by addition of 100 μL MH broth into all wells except for the first well of each row. For each inhibitor, 200 μL of the highest concentration was dissolved in MH broth (+ 10 μg/mL novobiocin) and was added into the first well of each row. The inhibitors used in this study and their highest concentrations were DNAseI (25 U/mL, Sigma), sodium (meta) periodate (4 mg/mL, Sigma), proteinase K (20 mg/mL, Promega), trypsin (0.05%, Gibco), H_2_O_2_ (30% pure, Sigma) and sodium deoxycholate (10%, Sigma). Using a multiwell pipettor, 100 μL of the medium in the first well of each row was diluted in a 1:2 serial dilution along the row to a final volume of 200 μL. The adherent biofilms were then incubated at 37 °C for 1 h in the presence of inhibitors. In order to remove non-adherent bacteria, the wells were then washed 3 times with PBS (Sigma). The metabolically active bacteria were then stained for 30 mins with PBS containing 40 μg/mL 5-TAMRA-SE (5-Carboxytetramethylrhodamine, Succinimidyl Ester, single isomer, ThermoFisher Scientific) viability stain followed by 3 washes with PBS [28, 29]. This rhodamine-based dye is cell permeant and upon entering the cytosol is enzymatically crosslinked by bacterial esterases, which prevents the rhodamine dye from escaping the intact bacterial cell, providing a bright indicator of living bacteria. Counterstaining of dead bacteria and extracellular DNA structures was subsequently carried out by staining the wells with PBS containing 10 μg/mL SytoX green dye for a further 30 min, followed by 3 additional PBS washing steps [31]. Upon staining with TAMRA/SytoX, the wells were washed with PBS a further 3 times followed by fixing with 4% PFA for 10 min. Automated confocal microscopy was carried out using an Opera Phenix (PerkinElmer) high-content screening microscope using a 5x/0.16 NA air objective. Images were acquired for channels using laser channels 561 nm (TAMRA) and 488 nm (SytoX). Images within all experiments were acquired with the same illumination and detection settings.

### Automated image analysis of adherent biofilm

A high-throughput image analysis approach for quantification of TAMRA and SytoX intensity and biofilm area was developed using the Columbus image data and analysis system v.2.8.2 (PerkinElmer). The building blocks involved in the analysis pipeline are described in supplementary Table 1. The output of the Columbus analysis resulted in a .csv file containing information of quantification of the mean biofilm area (px^2^) and intensity (AU) for each channel.

### Data processing and representation

**Fig. 1:**
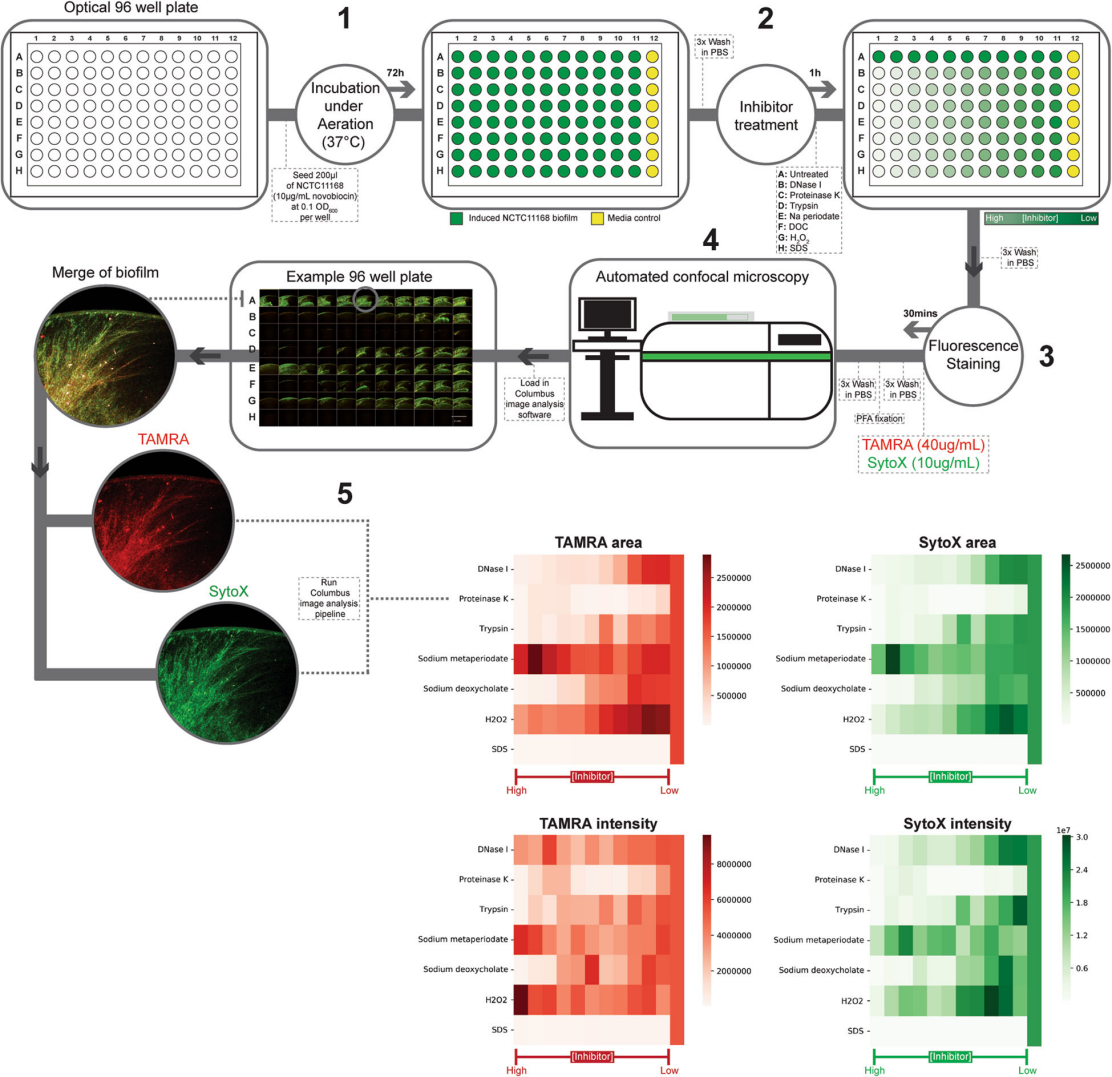
Workflow of high-content screening approach to investigate the effect of a range of inhibitors on the structure, composition and integrity of Campylobacter biofilm under aeration. (**1**) Bacterial overnight cultures of NCTC11168 (Induced with 10μg/mL novobiocin) were equalised to an OD600 of 0.1 in MH broth (supplemented with 10μg/mL novobiocin) and 200μL were seeded into all of the wells of an optical 96-well plate. The cultures were incubated for 72h at 37°C aerobically (21% O2) to induce adherent biofilm formation. (**2**) Each row of the 96-well plate was selected for addition of an inhibitor to be tested for its effect on biofilm integrity. After 72h incubation, the adherent biofilms in all 96 wells were washed once with PBS in aseptic conditions followed by addition of 100μL MH broth (+10μg/mL novobiocin) into all wells except for the first well of each row. For each inhibitor, 200μL of the highest concentration was dissolved in MH broth (+10ug/mL novobiocin) and was added into the first well of each row. The inhibitors used in this study and their highest concentrations were DNAseI (25U/mL, Sigma), sodium (meta)periodate (4mg/mL, Sigma), proteinase K (20mg/mL, Promega), trypsin (0.05%, Gibco), H2O2 (30% pure, Sigma) and sodium deoxycholate (10%, Sigma). Using a multiwell pipettor, 100μL of the medium in the first well of each row was diluted in a 1:2 serial dilution along the row to a final volume of 200μL. The adherent biofilms were then incubated at 37°C for 1h in the presence of inhibitors. (**3**) The metabolically active bacteria were stained for 30 mins with PBS containing 40μg/mL 5-TAMRA-SE followed by counterstaining of dead bacteria and extracellular DNA structures with PBS containing 10μg/mL SytoX green dye for a further 30 minutes. (**4**) Automated confocal microscopy was carried out using an Opera Phenix (PerkinElmer) high-content screening microscope using a 5x/0.16 NA air objective. Images were acquired for channels using laser channels 561nm (TAMRA) and 488nm (SytoX). (**5**) A high-throughput image analysis approach for quantification of TAMRA and SytoX intensity and biofilm area was developed using the Columbus image data and analysis system. Heatmaps of mean biofilm area intensities across three biological replicates were generated to provide a rapid readout of biofilm inhibition

Quantified adherent biofilm area (px^2^) and intensity (AU) for each inhibited biofilm were normalized to the mean uninhibited biofilm. Data are representative of three biological replicates per condition. Error bars represent the standard deviation for each sample condition. All graphs and statistical analysis were carried out in GraphPad Prism v.6.01 (GraphPad Software). Heatmaps of mean biofilm intensities and area in Fig. 1 were generated using the Python data visualization library ‘Seaborn’, based on matplotlib.

## Results

### Developing an inhibitor screening approach to elucidate the structural composition of induced aerobic biofilm

To allow for a more systematic approach to analyse adherent biofilm formation for larger sample sizes and more conditions, a high-content screening approach was developed as a modification to the biofilm analysis pipeline we developed previously [27]. Optical 96-well plates in which adherent biofilm had formed would proceed to be imaged using an Opera Phenix (PerkinElmer) high-content screening microscope using a 5x/0.16 NA air objective, to allow for a rapid acquisition of confocal images while minimizing the influence of user bias or error during image acquisition. In each 96-well plate, the adherent NCTC11168 biofilm phenotype was induced in 88 wells. Taking into account a positive control row of uninhibited biofilm and a column of MH broth blank, this allowed for the screening of seven biofilm inhibitors at 11 concentrations.

To gain insight into the molecular composition of the inducible NCTC11168 biofilm after 72 h incubation in high oxygen conditions, the structural integrity of the EPS component of the adherent biofilm was then challenged with the structural inhibitors chosen to disrupt eDNA (DNase I), EPS carbohydrate (Sodium (meta) periodate) and protein integrity (proteinase k, trypsin). Two biologically relevant compounds were also screened to represent intestinal bile salt stress (sodium deoxycholate), oxidative stress (H_2_O_2_). A detergent (sodium dodecyl sulphate) was selected as a positive control. A 1:2 dilution ratio was carried out to serially dilute the inhibitors in each well. Upon exposure to the inhibitors for 1 h, metabolically active biofilm was labelled with 5′-TAMRA-SE and dead cells and eDNA labelled with the nucleic acid dye SytoX. As the workflow in Fig. 1 demonstrates, rapid automated image acquisition was carried out using a PerkinElmer high-content screening confocal microscope. The top of the wells were imaged for each condition as this appeared to be the area in which uninhibited was biofilm consistently present during the PBS washing and aspirating steps. Image acquisition was carried out for TAMRA (568 nm) and SytoX (488 nm) channels. For this pilot screen, this resulted in 576,16-bit TIFF images, each averaging 8.9 MB in size, being produced.

Given the large data set, an automated analysis pipeline (Supplementary Table 1) was constructed using the Columbus (PerkinElmer) high-content image analysis software. Adherent biofilms were segmented automatically using a population-based thresholding of the TAMRA and SytoX signal. The fluorescence signal was then thresholded to exclude objects smaller than 4μm^2^ and the area in px^2^ and intensity (AU) of the biofilm quantified. Using the Seaborn matplotlib python package, a heatmap of the data was rapidly visualized to obtain immediate information about the effect of inhibitors on biofilm integrity (Fig. 1). For quantification of the area and intensity of inhibited TAMRA-stained and SytoX-stained biofilms, each biofilm was normalized to the mean of 11 uninhibited biofilms per replicate, with 1.0 representing an ‘uninhibited’ biofilm phenotype.

### The effect of DNase I and sodium metaperiodate on an induced aerobic biofilm

**Fig. 2:**
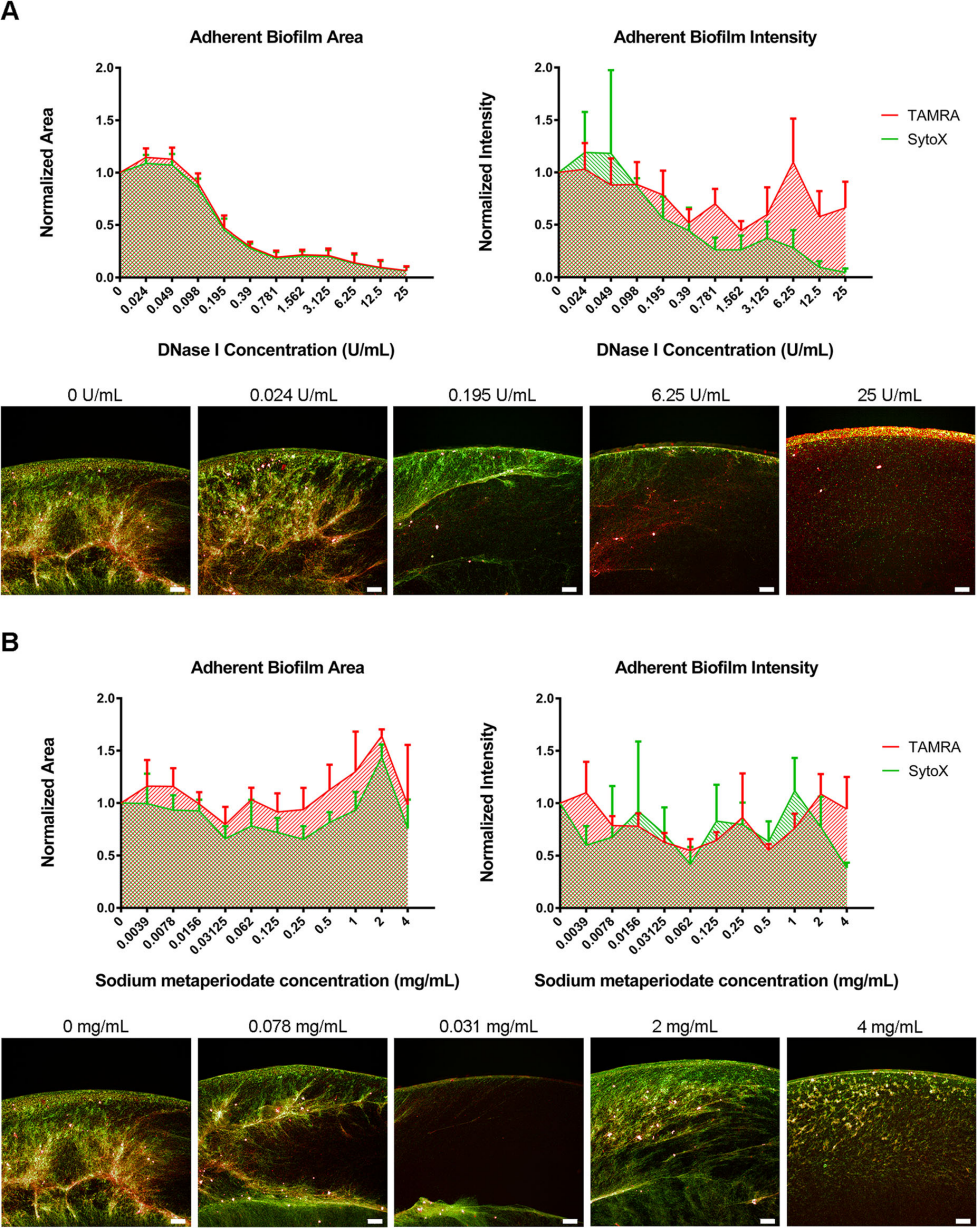
(**A**) Investigating the ability of DNase I to disrupt adherent biofilm of NCTC11168 induced with 10μg/mL novobiocin, under aeration. Quantification of TAMRA and SyoX labelled adherent biofilm area (px2) (**Left**) and intensity (AU) (**Right**), normalised to uninhibited biofilm. (**Bottom**) Images of TAMRA labelled (red) and SytoX (green) labelled biofilm post-incubation with DNase I. (**B**) Investigating the ability of sodium (meta) periodate to disrupt adherent biofilm of NCTC11168 induced with 10μg/mL novobiocin, under aeration. Quantification of TAMRA and SyoX labelled adherent biofilm area (px2) (**Left)** and intensity (AU) (**Right**), normalised to uninhibited biofilm. (**Bottom**) Images of TAMRA labelled (red) and SytoX (green) labelled biofilm post-incubation with sodium periodate. Data shown is mean of three replicates per condition +/- SD, n=3. Scale bar = 100µm

As Fig. 2a highlights, as the concentration of DNase I increase there was a dramatic loss of biofilm area with a visible change in eDNA structure (SytoX, green) observable at the low concentration of 0.025 U/mL, which supports our previous observations of the dense eDNA matrix observed [27]. Interestingly, metabolically active bacteria (TAMRA) intensity within the biofilm remained structurally intact at higher concentration even after dissolution of the eDNA matrix, indicating that despite destruction of the biofilm ultrastructure, a significant proportion of bacteria remained adherent and viable.

Sodium (meta) periodate caused minimal observable disruption to the area of TAMRA or SytoX biofilm, even at concentrations as high as 4 mg/mL. Interestingly, at high concentrations of sodium (meta) periodate, the structure of the biofilm appeared to have changed with a visibly less dense matrix (Fig. 2b). This was further supported by a decrease in SytoX positive biofilm intensity when exposed to 4 mg/mL sodium (meta) periodate.

### The effect of proteases on an induced aerobic biofilm

**Fig. 3:**
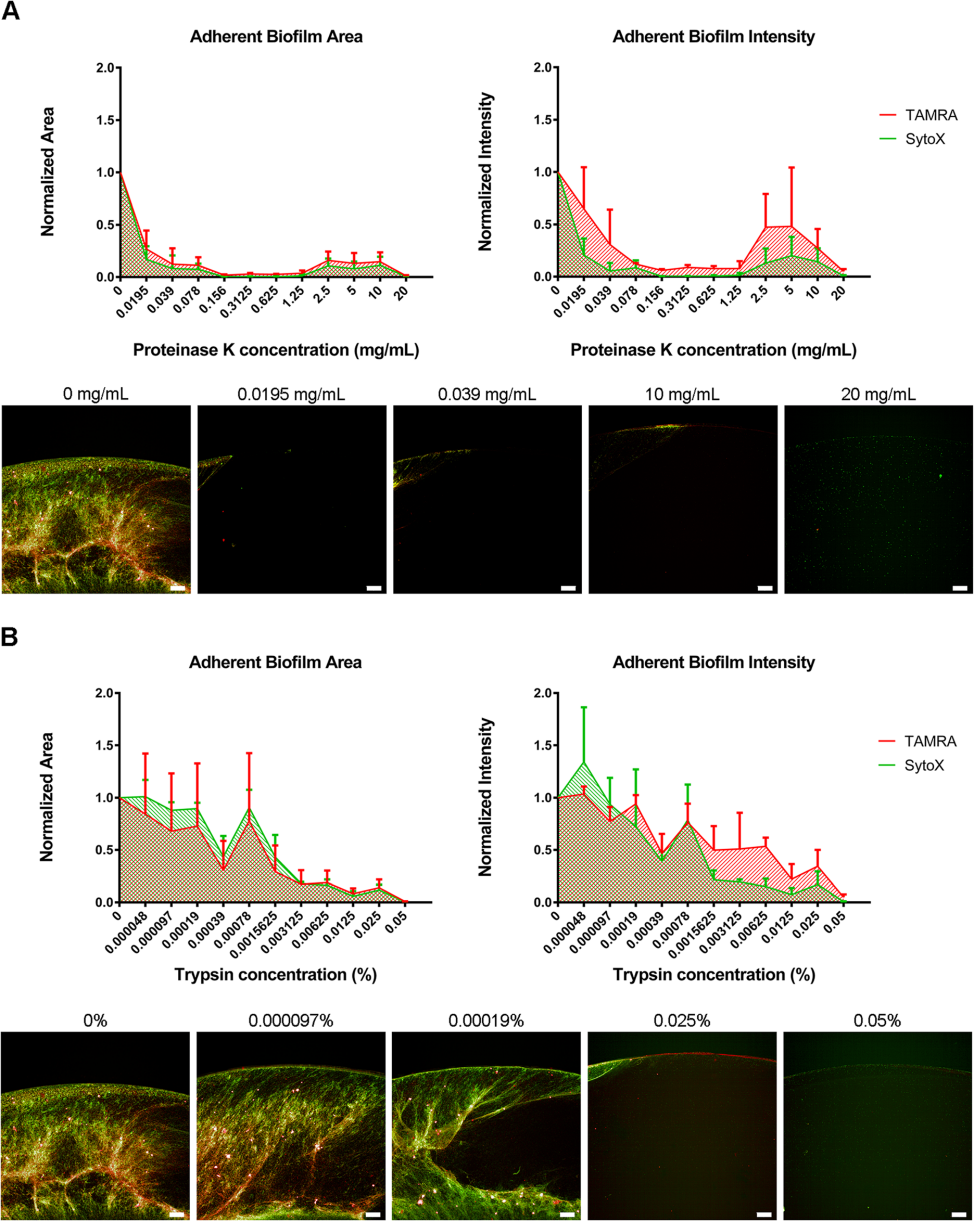
(A) Investigating the ability of proteinase K to disrupt adherent biofilm of NCTC11168 induced with 10μg/mL novobiocin, under aeration. Quantification of TAMRA and SyoX labelled adherent biofilm area (px2) (Left) and intensity (AU) (**Right**), normalised to uninhibited biofilm. (**Bottom**) Images of TAMRA labelled (red) and SytoX (green) labelled biofilm post-incubation with Proteinase k. (B) Investigating the ability of trypsin to disrupt adherent biofilm of NCTC11168 induced with 10μg/mL novobiocin, under aeration. Quantification of TAMRA and SyoX labelled adherent biofilm area (px2) (**Left**) and intensity (AU) (**Right**), normalised to uninhibited biofilm. (**Bottom**) Images of TAMRA labelled (red) and SytoX (green) labelled biofilm post-incubation with trypsin. Data shown is mean of three replicates per condition +/- SD, n=3. Scale bar = 100µm

Proteinase K and trypsin, which are both serine proteases were very effective at disrupting the integrity of the biofilm (Fig. 3a + b). Proteinase K was able to dramatically disrupt the inducible biofilm area at concentrations as low as 0.0195 mg/mL and remove the entire metabolically active NCTC11168 TAMRA intensity signal at 0.078 mg/mL. Trypsin, which was screened at lower concentrations than proteinase K, followed a very similar profile of disruption of SytoX/TAMRA biofilm area and intensity. At concentrations of 0.001% the trends of disruption of the two biofilm stains differed with the eDNA/DNA stained biofilm being totally disrupted while a proportion of the metabolically active (TAMRA) cells remained adherent (Fig. 3a).

### The effect of environmental stressors sodium deoxycholate and hydrogen peroxide on induced aerobic biofilm integrity

The ability of a viable biofilm to survive and maintain structural integrity under two environmental stressors that would be encountered during *C. jejuni* pathogenesis from the farm to the jejunum was next determined. Firstly, the antimicrobial bile salt sodium deoxycholate, which is encountered in the intestine was selected [32, 33]. Secondly, H_2_O_2_ which promotes the oxidative stress response already observed during aeration, in the phagosome and also may play a role in the intestinal epithelium in mucosal defence against microbial pathogens [34]. The detergent sodium dodecyl sulphate (SDS) was also used as a positive control for total disruption of the biofilm.

**Fig. 4:**
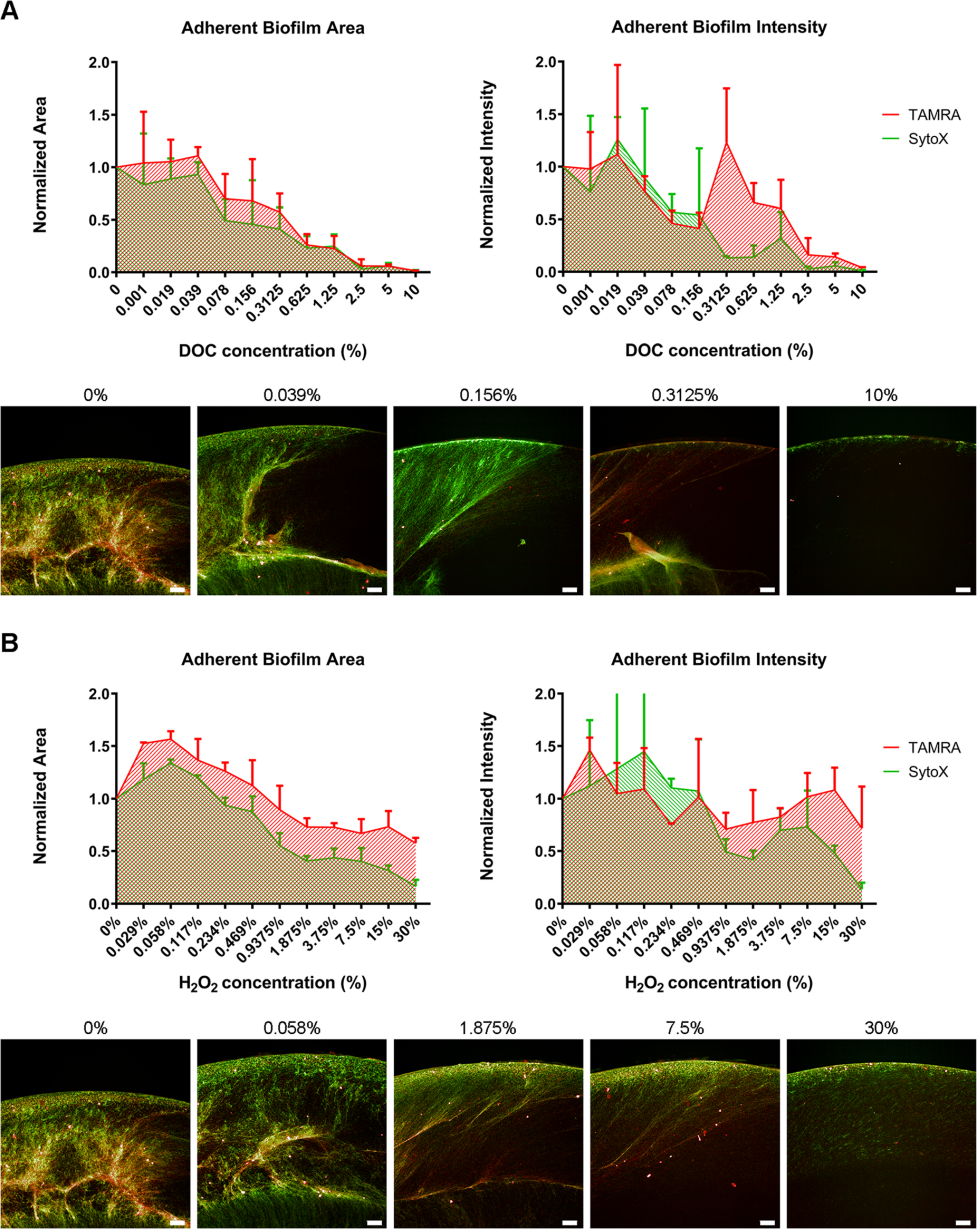
(A) Investigating the ability of sodium deoxycholate (DOC) to disrupt adherent biofilm of NCTC11168 induced with 10μg/mL novobiocin, under aeration. Quantification of TAMRA and SyoX labelled adherent biofilm area (px2) (Left) and intensity (AU) (**Right**), normalised to uninhibited biofilm. (**Bottom**) Images of TAMRA labelled (red) and SytoX (green) labelled biofilm post-incubation with DOC. (B) Investigating the ability of H2O2 to disrupt adherent biofilm of NCTC11168 induced with 10μg/mL novobiocin, under aeration. Quantification of TAMRA and SyoX labelled adherent biofilm area (px2) (**Left**) and intensity (AU) (**Right**), normalised to uninhibited biofilm. (**Bottom**) Images of TAMRA labelled (red) and SytoX (green) labelled biofilm post-incubation with H2O2. Data shown is mean of three replicates per condition +/- SD, n=3. Scale bar = 100µm

NCTC11168-induced biofilm structure was significantly disrupted by concentrations of sodium deoxycholate at concentrations of 0.3125% w/v. However, a visible trend of disruption was evident at 0.078% w/v for both TAMRA and SytoX positive biofilm (Fig. 4a). This was interesting as the physiologically relevant concentration of sodium deoxycholate in the human intestine is 0.1% w/v [19].

As this NCTC11168 biofilm was induced specifically under aeration, the NCTC11168 biofilm was highly resistant to 1 h exposure to H_2_O_2_. At low concentrations of H_2_O_2_, 0.029–0.117% for SytoX, and 0.029–0.234% for TAMRA, a significant increase in biofilm area compared to the uninhibited biofilm was observed.

Disruption of both TAMRA positive and SytoX positive biofilm area was dramatic after 1 h incubation with 1.8765% H_2_O_2_. However, clusters of metabolically active cells were still observed to be attached at concentration of 7.5% or higher (Fig. 4b) which was evidenced by the relative lack of disruption of TAMRA signal intensity at high concentrations of H_2_O_2_. Treatment of the induced biofilm with the detergent sodium dodecyl sulphate (SDS) resulted in total disruption of both adherent bacteria and eDNA matrix components at concentrations as low as 0.029%. This clearly Indicates the power of surfactants at removal of *C. jejuni* adherent biofilm from an abiotic surface (Supplementary Figure 1).

## Discussion

A significant challenge in the field of *Campylobacter* biofilm research has been the low-throughput of traditional imaging based methodological approaches and biofilm heterogeneity amongst strains in which much of the work has been done to investigate the core EPS composition. The uniformity of adherent biofilm produced by the induced aerobic biofilm phenotype shown in our previous work [27] offered a unique opportunity to robustly screen large numbers of homogenous *Campylobacter* biofilms using a variety of relevant inhibitors. Biofilm formation under aeration was of particular interest, as high oxygen levels are the most prominent environmental stressor experienced by *C. jejuni* residing on chicken carcasses, during the transmission to humans. Indeed, high levels of available oxygen are capable of relaxing negative supercoiling of bacterial DNA. Bacterial pathogens such as *S. typhimurium* take advantage of supercoiling sensitive regulators of gene expression to express cohorts of genes conferring the tools necessary to survive in oxidative stress inducing environments such as intracellular environment of macrophages [35].

An advantage of the development of the high-content screening approach described here to assess adherent biofilm formation is that it was possible to robustly screen for inhibitors of the biofilm induced by relaxation of DNA supercoiling under aeration. A significant advantage of this approach is that it allowed for a large number of positive controls, 33 uninhibited biofilms, to be assessed. This therefore provided a robust quantification of the baseline size and intensity of *Campylobacter* biofilm. The use of a fluorescent labelling system allowed for the quantification of the effect of biofilm size (area) and density (intensity) for the metabolically active living bacterial population (TAMRA) as well as the largely eDNA EPS component of the biofilm (SytoX).

A pilot screen was conducted to broadly investigate the EPS structural composition of the induced biofilm of NCTC11168 by treatment with increasing concentrations of DNase I (degradation of eDNA), sodium (meta) periodate (oxidisation/ disruption of carbohydrate), proteinase K and trypsin (serine protease mediated protein cleavage). As expected, degradation of eDNA led to efficient disruption of the induced biofilm between the concentrations of 0.39 U/mL to 25 U/mL. This was in agreement with the observations of Brown and colleagues [24]. However, they found that biofilm inhibition was observed at concentrations of DNase I of 0.01 U/mL which may indicate that an increase in eDNA matrix amount and density plays a significant role in this inducible biofilm phenotype. Interestingly, when incubated with 0.39 U/mL DNase I, a large proportion of biofilm remained adherent to the surface, while SytoX staining of DNA was largely abolished (Fig. 2a). A carbohydrate moiety did not appear to be present within the EPS of the induced biofilm as it was insensitive to treatment with sodium (meta) periodate, maintaining biofilm integrity and viability throughout treatment, which was in agreement with the observations of Melo and colleagues (2017) [26]. Interestingly, at high concentrations (4 mg/mL), sodium (meta) periodate, the 3D ultrastructure of the adherent biofilm changed in appearance (Fig. 2b). This was correlated with a sharp decline in the SytoX positive biofilm moiety. It is likely that disruption of surface glycans and possibly capsular protein glycosylation was to blame for this change in structure, however further work would have to be carried out to confirm this.

In agreement with the observations of the key role of bacterial adhesion in the relaxation of DNA supercoiling induced biofilm, both proteinase K (≥19.5 μg/mL) and trypsin (≥0.00039%) were highly efficient at disrupting the induced biofilm, likely through disruption of cell-cell/cell-surface adhesion mediated by the flagella and OM adhesins. These observations were also in line with the findings of Melo and colleagues (2017), who found that proteinase K was highly efficient at disrupting *C. jejuni* biofilm at 1 mg/mL. They suggested that costs of production are the main limiting factor in the use of proteinase K in biotreatment of *C. jejuni* biofilms in an agricultural setting, however the findings in this study suggest that proteinase K is highly efficient as clearing *C. jejuni* biofilm at very low treatment doses. Taken together, these findings are largely in agreement with the current literature, further supporting that this supercoiling induced biofilm is structurally representative of biofilms naturally formed by *C. jejuni*.

Three inhibitors representing ‘environmental stressors’ were screened for their ability to disrupt the novobiocin-induced biofilm phenotype of NCTC11168, the bile salt sodium deoxycholate (DOC), H_2_O_2_ and sodium dodecyl sulphate (SDS). Firstly, DOC has been shown to induce a highly secretory invasive phenotype in *C. jejuni* [19]. DOC has also been shown to enhance biofilm formation with incubation of strain 81–176 in the presence of 0.05% DOC promoting adherent biofilm formation, autolysis and eDNA release [1]. Interestingly, no increase in adherent biofilm formation at ‘subinhibitory’ concentrations of DOC treatment was observed (Fig. 4a). At concentrations of 0.3125% DOC and above, disruption of the adherent biofilm occurred. It is possible that the increase observed by Svensson and colleagues (2014) occurs specifically when incubated microaerobically, or there is a possibility that the novobiocin-induced phenotype represents the ‘maximum’ biofilm possible by this number of bacteria, therefore being insensitive to the regulatory triggers of DOC. Interestingly, at concentrations of 0.3125–1.25% DOC treatment, remediation of the inhibition of TAMRA positive biofilm intensity was observed. Indicating that under these conditions there was a significant proportion of the metabolically active cells within the population remaining adherent to the well.

Treatment with H_2_O_2_ was utilised to investigate maintenance of biofilm integrity in oxidative stress coupled with aerobic stress. Interestingly, the novobiocin-induced biofilm was uninhibited until treatment with 0.9376% H_2_O_2_ or higher (Fig. 4b). It was notable that at very low levels of dissolved H_2_O_2_ (0.029 and 0.058%), there was an increase in viable biofilm in addition to the novobiocin-induced biofilm. This could be explained by the further relaxation of DNA supercoiling homeostasis by ROS at subinhibitory levels. This observation supports the hypothesis that this high oxygen abundance acts as a major environmental driving factor to promote global upregulation of genes involved in biofilm formation, invasion and oxidative stress response in *C. jejuni*. The observation that viable (red) bacteria were present after incubation in levels of 7.5% H_2_O_2_ or greater (up to 30%) appear to be higher than the H_2_O_2_ tolerance levels reported for *C. jejuni* biofilm in the literature [36]. This indicates that this viable biofilm would not only provide benefits to host defence reactive oxygen species but also promote transmission via survival in the high oxygen environment of the supermarket. This could also confer advantages to survival of H_2_O_2_-mediated cleaning using 10–30% H_2_O_2_ resulting in less efficient killing of the *C. jejuni*. Interestingly, the *ΔperR* mutant is capable of surviving in far higher levels of H_2_O_2_ than WT *C. jejuni*, due to the derepression of oxidative stress response genes controlled by the PerR regulon [36, 37]. It is reasonable to suggest that relaxation of DNA supercoiling, which is induced by presence of oxygen, can upregulate the secretion of oxidative stress response genes which in conjunction with modification of the flagella and increasing OMP adhesins, promote biofilm formation in aerobic conditions.

In the past, high-content screening approaches have predominantly been utilised in antimicrobial drug screening when implemented in the context of microbial pathogenesis. In this work we present a novel imaging-based biofilm screening platform. This methodology could provide significant advantages in the rapid testing of the ability of large numbers of *C. jejuni* strains to form biofilm in a variety of environmental contexts, as well as to dissect the molecular mechanisms at play during *C. jejuni* biofilm formation. This approach also offers the opportunity to be easily adapted to phenotypically screen and characterise adherent biofilm of large numbers of fresh bacterial isolates of a variety of bacterial pathogens. In the modern era of big ‘omic’ data in relation to genomic, transcriptomics and proteomics, approaches such as the one described here offer an opportunity to study biofilm formation by pathogens on a much larger scale.

## Supplementary Information

**Additional file 1.** Automated image analysis pipeline as executed using the PerkinElmer Columbus image analysis suite. **Figure 1**. (**A**) Investigating the ability of sodium dodecyl sulphate (SDS) to to disrupt adherent biofilm of NCTC11168 induced with 10μg/mL novobiocin, under aeration. Quantification of TAMRA and SyoX labelled adherent biofilm area (px2) (**Left**) and intensity (AU) (**Right**), normalised to uninhibited biofilm. (**Bottom**) Images of TAMRA labelled (red) and SytoX (green) labelled biofilm post-incubation with SDS. Data shown is mean of three replicates per condition +/- SD, n=3. Scale bar = 100µm.
