# Supplementary material for: A novel high-content screening approach for the elucidation of C. jejuni biofilm composition and integrity
Source: BMC Microbiol. 2021 Jan 4;21:2. doi: 10.1186/s12866-020-02062-5 (PMC7784365; doi:10.1186/s12866-020-02062-5)

A novel high-content screening approach for the elucidation of *C. jejuni* biofilm composition and integrity

Matthew V.X. Whelan[1], Jeremy C. Simpson[2] and Tadhg Ó Cróinín[1]*

Supplementary figures

Supplementary table 1.

Columbus analysis pipeline			
**Input Image**	**Input**		
	Flatfield Correction: None<br>Stack processing: Individual Planes<br>Min. Global Binning: Dynamic		
**Find Cells**	**Input**	**Method**	**Output**
	Channel: Alexa 568<br>ROI: None	Method: P<br>Area: > 4µm²<br>Splitting Sensitivity: 0.5<br>Common Threshold: 0.55	Output Population:<br>TAMRA
**Calculate Intensity Properties**	**Input**	**Method**	**Output**
	Channel: Alexa 568<br>Population: TAMRA<br>Region: Cell	Method: Standard<br>Mean	Property Prefix:<br>Intensity TAMRA
**Calculate Morphology Properties**	**Input**	**Method**	**Output**
	Population: TAMRA<br>Region: Cell	Method: Standard<br>Area<br>Roundness	Property Prefix:<br>Biofilm TAMRA
**Find Cells**	**Input**	**Method**	**Output**
	Channel: Alexa 488<br>ROI: None	Method: P<br>Area: > 4µm²<br>Splitting Sensitivity: 0.5<br>Common Threshold: 0.55	Output Population:<br>SytoX
**Calculate Intensity Properties**	**Input**	**Method**	**Output**
	Channel: Alexa 488<br>Population: SytoX<br>Region: Cell	Method: Standard<br>Mean	Property Prefix:<br>Intensity SytoX
**Calculate Morphology Properties**	**Input**	**Method**	**Output**
	Population: SytoX<br>Region: Cell	Method: Standard<br>Area<br>Roundness	Property Prefix:<br>Biofilm SytoX
**Define Results**	**Results**		**Object Results**
	Method: list of outputs<br>**Population: TAMRA**<br>Number of Objects<br>Apply to All: ALL<br>Intensity TAMRA Mean: ALL<br>Biofilm TAMRA Area [px²]: ALL<br>Biofilm TAMRA Roundness: ALL	Method: list of outputs<br>**Population: SytoX**<br>Number of Objects<br>Apply to All: ALL<br>Intensity SytoX Mean: ALL<br>Biofilm SytoX Area [px²]: ALL<br>Biofilm SytoX Roundness: ALL	Population: TAMRA: ALL<br>Population: SytoX: ALL

Supplementary figure 1.

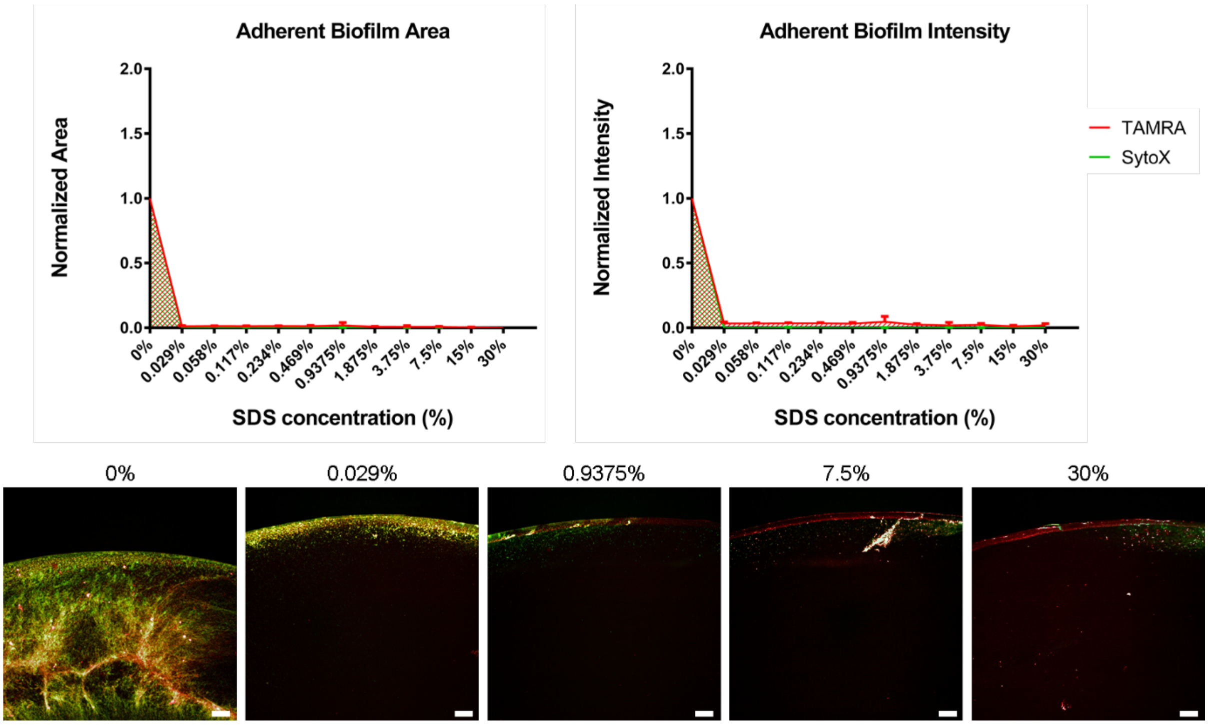

Supplement: Supplementary file 1 — Additional file 1. Automated image analysis pipeline as executed using the PerkinElmer Columbus image analysis suite. Figure 1. (A) Investigating the ability of sodium dodecyl sulphate (SDS) to to disrupt adherent biofilm of NCTC11168 induced with 10μg/mL novobiocin, under aeration. Quantification of TAMRA and SyoX labelled adherent biofilm area (px2) (Left) and intensity (AU) (Right), normalised to uninhibited biofilm. (Bottom) Images of TAMRA labelled (red) and SytoX (green) labelled biofilm post-incubation with SDS. Data shown is mean of three replicates per condition +/- SD, n=3. Scale bar = 100µm. [file 12866_2020_2062_MOESM1_ESM.pdf]
